# Hepatopulmonary hydatidosis in a ten-year-old girl: a case report

**DOI:** 10.1186/1752-1947-4-205

**Published:** 2010-07-02

**Authors:** Dimitrios Anyfantakis, Evangellos Blevrakis, Ioannis Vlachakis, Ioannis Arbiros

**Affiliations:** 1 Department of Pediatric Surgery, University General Hospital of Heraklion, Crete, Greece

## Abstract

**Introduction:**

Hydatid disease is a parasitic infection caused by the tapeworm *Echinococcus granulosus *and is characterised by cystic lesions in the liver and lungs. Concomitant pulmonary and hepatic localization of hydatid cysts in childhood is unusual and represents a distinct clinical entity called hepatopulmonary hydatidosis.

**Case presentation:**

A ten-year-old Caucasian girl, a permanent resident of rural Greece, was admitted to hospital reporting a nonspecific symptomatology compatible with a diagnosis of viral infection. Chest radiography revealed a large homogenous circular opacity in the right lung field. On the basis of imaging studies, a diagnosis of hydatidosis was made with synchronous hepatic and pulmonary involvement, successfully managed through a single-stage transthoracic surgical approach.

**Conclusion:**

This case report highlights the necessity of realizing that hydatid disease continues to be a public health problem, which often remains asymptomatic for years. Therefore, the presence of any homogeneous cystic spherical opacity on routine chest radiography should raise the suspicion of hydatid disease, mainly in endemic areas such as Greece. General practitioners and physicians involved in pediatric care need to be familiar with the diagnosis and management of the variable clinical manifestations of hydatid disease. Taking into consideration that hepatopulmonary hydatidosis represents a special entity that requires a different therapeutic approach may positively affect its economic and social-related burden.

## Introduction

Hydatid disease is caused by the tapeworm *Echinococcus granulosus *[1] and represents the most important parasitic infection in the Mediterranean region [2]. Greece, in particular, is a highly endemic area, partly because of the family character of occupation with animal husbandry observed in rural areas [2]. The liver is the most commonly affected organ (> 65%) followed by the lung (> 25%) [1], which is the predominant site of cyst formation in children. We report a case with concomitant hepatic and pulmonary hydatid disease in a 10-year-old girl.

## Case presentation

A ten-year-old Caucasian girl, a permanent resident of rural Crete, Greece, was admitted to hospital reporting fever, anorexia and fatigue that had been present for the previous four days. Her vital signs were as follows: body temperature, 37.8°C; blood pressure, 120/60 mm Hg; heart rate, 95 beats/min; and respiratory rate, 12 breaths/min. The oxygen saturation was 96% while she was breathing ambient air. Pulmonary examination revealed decreased breath sounds and dullness to percussion over the right costophrenic angle. The rest of the physical examination results were normal. Laboratory investigations included a complete blood count, liver function tests and urine analysis; all results were within normal ranges except for 3% eosinophils on the white blood cell differential.

**Figure 1:**
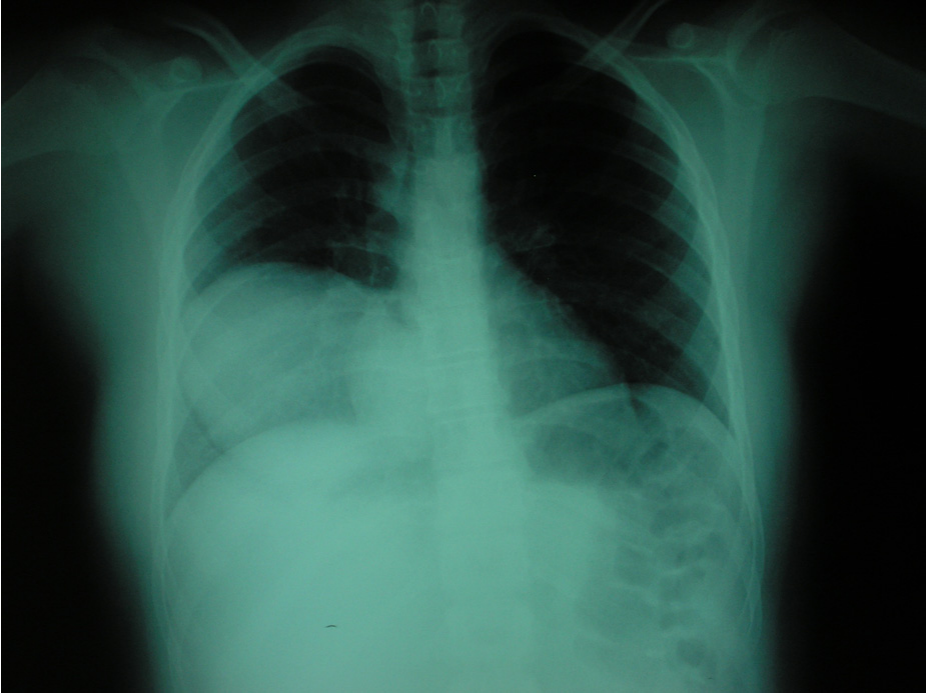
Chest radiograph shows a 9.0 × 8.2 cm homogenous circular opacity in the lower lobe of the right lung.

**Figure 2:**
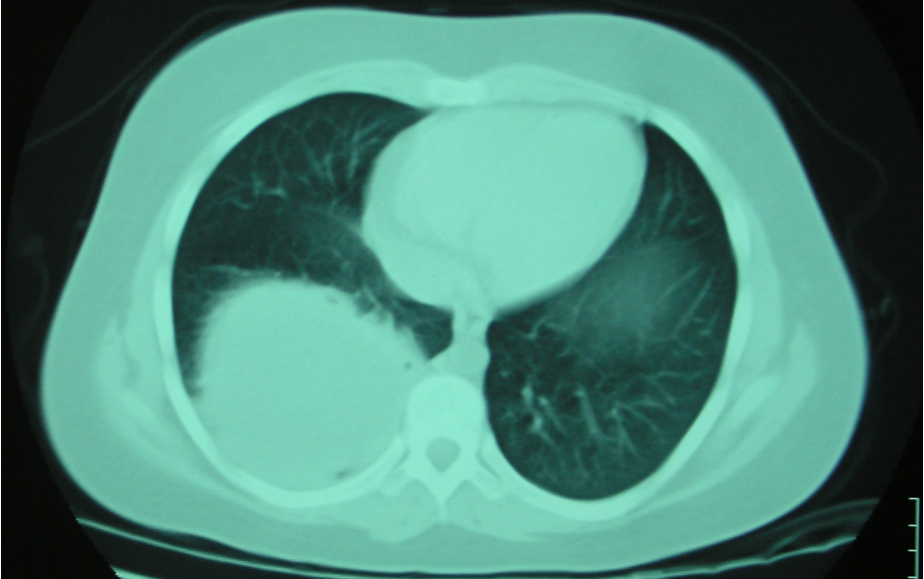
Computed tomography thoracic scan reveals the presence of a large cystic lesion in the right lower lobe.

**Figure 3:**
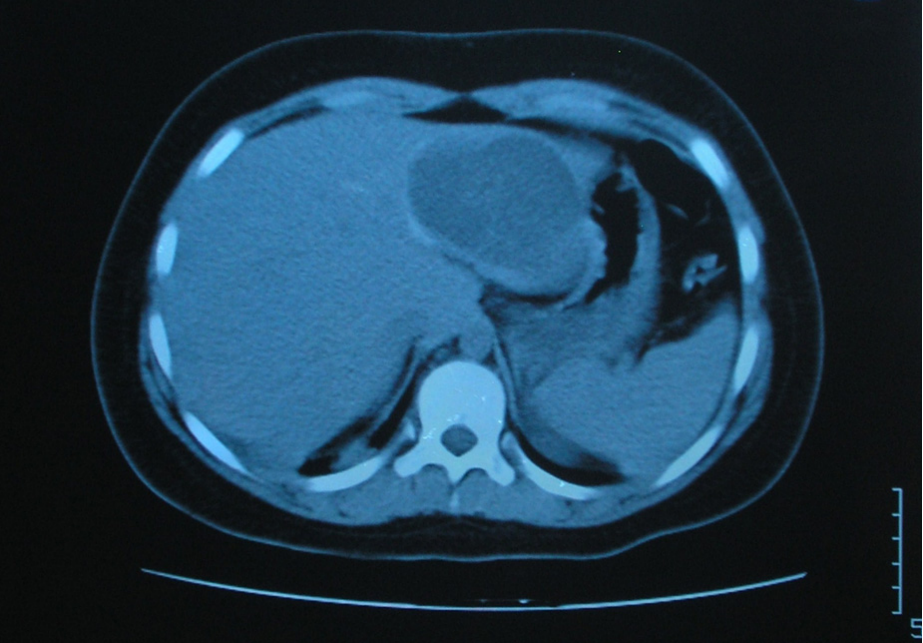
Computed tomography abdominal scan shows a cystic lesion in the left lobe of the liver.

On chest radiography, a 9.0 × 8.2 cm circular lesion was visualized in the right lower lobe (Figure 1). A computed tomography (CT) thoracic scan revealed a cystic fluid-filled mass, 9.0 cm in diameter, on the inferior lobe of the right lung (Figure 2). The girl's history of living in proximity to dogs and farm animals was suggestive of infection with *E. granulosus*. The eventuality of liver involvement was investigated; a CT abdominal scan examination revealed the presence of a hypodense cystic lesion of 7.9 × 7.2 cm in the left lobe of the liver (Figure 3). Serologic test results for echinococcus by means of an enzyme-linked immunosorbent assay and an indirect haemagglutination test were positive.

A single-stage surgical approach was considered. Surgical excision of the pulmonary cyst was performed through a right posterolateral thoracotomy. After identification of the cyst, the lung was surrounded by sterile gauze sponges to minimize the risk of spillage of daughter vesicles into the thoracic cavity. A needle connected to the suction tip was carefully inserted into the cystic cavity to aspirate the cystic fluid. After the evacuation of the cystic contents, the most superficial part of the cyst was opened (cystotomy), and the laminated membrane was removed with ring forceps. The remaining pulmonary cavity was irrigated with saline solution and cleaned with sterile gauze sponges. Then the bronchial openings were sutured, and the residual cavity was obliterated by absorbable pursestring sutures starting from the deepest level to the surface. After surgery of the pulmonary cyst, the diaphragm was incised radially over the palpable liver cyst. Aspiration of the cystic contents was performed by a large-bore needle followed by cystotomy and careful removal of germinative membrane. Because there was no bile leak on the inner surface of the cyst, the residual cavity and the remnant pericystic hepatic tissue were inverted with sutures of Vicryl. A catheter was positioned in the residual cavity below the diaphragm. Histopathologic examination of the cysts confirmed the diagnosis of hydatid disease. The girl's postoperative period was uneventful. The subdiaphragmatic and chest drains were removed on the fourth and sixth postoperative day, respectively. The girl was discharged on the eighth day after surgery. Adjunctive chemotherapy with oral albendazole (400 mg twice daily) was administrated for two months to prevent recurrence. During the 12-month follow-up with four-month intervals of ultrasonography and chest radiography, there was no evidence of recurrence.

## Discussion

Human echinococcosis was first described in ancient times by Hippocrates, as "cysts full of water" in a human liver [2]. In Greece, although after the 1970s morbidity rates of hydatid disease decreased, it still remains a public health problem with considerable impact on social and financial levels in terms of disability and cost [2]. Remarkably, for the period from 1967 to 1981, the incidence of hydatid disease was almost 13 cases per 100,000 inhabitants.

Diagnosis is based on clinical signs, laboratory results and epidemiologic data [3]. The combined use of ultrasonography and immunodiagnosis makes possible the distinction of echinococcal cysts from benign cysts, abcesses or malignant neoplasms [1]. Among serologic tests, immunoelectrophoresis has been reported to be the most specific for the primary diagnosis and postsurgical follow-up [4], and enzyme-linked immunosorbent assay (ELISA) represents a valuable diagnostic test for the initial screening [1]. However, the sensitivity of these tests is limited by the high percentage of false-negative results (in up to one of four patients) [3]. Despite the advances made in imaging techniques, the diagnosis of hydatid disease remains a challenging issue [3]. The diagnostic delay is partially attributed to the slow growth rate of hydatid cysts (from 1 to 5 cm in diameter annually) remaining well-tolerated by patients for long periods [1]. Remarkably, although echinococcal infections may be acquired during childhood, a maximum two of 10 patients are diagnosed before age 16 years of age [1]. Currently, hydatid disease is classified among the most neglected parasitic diseases [5].

For many years, surgery was the traditional treatment of choice for hydatid disease [1]. However, the modern management involves new treatment modalities and varies from surgical intervention to percutaneous drainage or chemotherapy with benzimidazole compounds [1]. A minimally invasive percutaneous procedure, introduced in the past two decades, was developed as an attractive alternative to surgery and consists of *p*ercutaneous treatment by puncture of the cyst, *a*spiration of cyst fluid, *i*njection of a scolicidal agent, and *r*easpiration of the cyst contents (PAIR) under sonographic guidance [5]. The aim of the PAIR treatment is to destroy the germinal layer with scolicidal agents or to evacuate the entire endocyst [5]. It is indicated for patients with single or multiple cysts in the liver, spleen, kidney and bones and for those who cannot undergo surgery [1]. PAIR represents an effective, minimally invasive and safe procedure [3] with a low rate of complications [1] and is the only method that provides direct diagnostic information regarding the parasitic nature of the cysts [3]. Complications, although rare, include secondary infection of the cavity caused by spillage of the cystic fluid, allergic reactions and recurrence [1]. PAIR is contraindicated for inaccessible or superficially located hepatic cysts, for inactive or calcified cystic lesions and in patients whose cysts have biliary communication [1]. Furthermore, application of PAIR treatment is not recommended for pulmonary cysts [1]. Other minimally invasive techniques, such as surgical procedure through a video-assisted thoracoscopy [6] or laparoscopy, have recently been proposed in selected patients [7].

Another widely used option in the management of hydatid disease, especially for inoperable cases, is chemotherapy with albendazole or mebendazole [3]. The minimum duration of treatment is three months [1]. A complete cure in one of three patients treated with benzimidazole drugs has been reported [1]. Furthermore, moderate evidence supports that chemoprophylaxis with antihelminthic medications during perioperative and postsurgical periods (up to three months) may reduce intraoperative complication and recurrence rates [5]. The efficacy of chemoprophylaxis in protection of humans living in endemic areas of hydatid infection represents an issue that remains to be established on the basis of methodologically reliable randomized, controlled trials [5].

Synchronous occurrence of pulmonary and hepatic hydatid cysts is an uncommon manifestation of hydatid disease that is observed in less than 10% of cases [8]. It represents a distinct clinical entity named *hepatopulmonary hydatidosis *(HPH). HPH typically does not occur in childhood. The condition is usually noticed in female patients, particularly in those older than 40 years of age [9]. Furthermore, contrary to this case, pulmonary cysts have a tendency to be bilateral and multiple [9]. The most frequent complaints reported in symptomatic patients with HPH are from the respiratory system, including cough, chest pain, dyspnea and hemoptysis [9]. In this case, the girl's clinical presentation was atypical. The diagnostic suspicion of hydatid disease was raised after the incidental visualization of the homogeneous spherical opacity on the chest radiographs and the history of frequent contact with dogs. HPH can cause greater economic and social burden compared with hydatidosis with single-organ involvement because the management of the former may require numerous surgical interventions and prolonged postoperative care. Therefore, appropriate diagnosis and an optimal surgical approach in this group of patients are issues of paramount importance [9]. A combined resection of hydatid cysts at both the sites during the same operation when feasible with a maximum preservation of the parenchyma in uncomplicated cysts (regardless of their dimension) appears to be a safe and optimal approach [9,10].

## Conclusion

This case report highlights the necessity of maintaining a high level of vigilance because hydatid disease is still an existent public health problem of worldwide significance that may remain asymptomatic and undiagnosed for a long period [1]. Awareness of the reemerging character of hydatid disease by general practitioners in particular, who often encounter a wide range of unspecific symptoms in their daily practice, may allow for early diagnosis. Early recognition is important because prompt intervention represents the key issue for the management of the disease [4]. Therefore, in endemic regions such as Greece, a high index of suspicion for hydatid disease is required by physicians, especially when circular cystic lesions are occasionally visualized on routine chest radiography.

Additionally, because there are many potential sites of cyst formation and the presence of multifocal disease affects the therapeutic strategy, it is necessary that patients with suspected pulmonary hydatid disease be investigated for the possibility of hepatic involvement [9]. Furthermore, physicians need to be familiar with the diagnosis and management of patients with the variable clinical manifestations of hydatid disease, such as HPH. Understanding that HPH represents a separate clinical entity that requires a different surgical approach may result in beneficial outcomes in terms of reducing its financial and occupational loss. In alignment with previous studies [9,10], the single-session surgical approach performed in our case for removal of pulmonary and hepatic hydatid cyst was safe and effective.

## Consent

Written informed consent was obtained from the parents of the child for publication of this case report and accompanying images. A copy of the written consent is available for review by the Editor-in-Chief of this journal.

## Competing interests

The authors declare that they have no competing interests.

## Authors' contributions

DA conceived the idea and drafted and prepared the manuscript. EB, IV and IA performed the operation and carried out the review of the patient's medical record to collect all the available information. EB, IV and IA provided clinical details and technical input, and DA revised the manuscript and performed editing and format changes throughout the manuscript. All authors have read and approved the final manuscript.
